# Extravascular lung water in patients with severe sepsis: a prospective cohort study

**DOI:** 10.1186/cc3025

**Published:** 2005-01-11

**Authors:** Greg S Martin, Stephanie Eaton, Meredith Mealer, Marc Moss

**Affiliations:** 1 Director, Medical and Coronary Intensive Care Units, Grady Memorial Hospital, and Assistant Professor of Medicine, Division of Pulmonary, Allergy and Critical Care, Emory University School of Medicine, Atlanta, Georgia, USA; 2 Grady Memorial Hospital, Emory University School of Medicine, Division of Pulmonary and Critical Care Medicine, Atlanta, Georgia, USA; 3 Senior Research Coordinator, Division of Pulmonary, Allergy and Critical Care, Emory University School of Medicine, Atlanta, Georgia, USA; 4 Section Head, Pulmonary Allergy and Critical Care Medicine, Grady Memorial Hospital, and Associate Professor of Medicine, Division of Pulmonary, Allergy and Critical Care, Emory University School of Medicine, Atlanta, Georgia, USA

## Abstract

**Introduction:**

Few investigations have prospectively examined extravascular lung water (EVLW) in patients with severe sepsis. We sought to determine whether EVLW may contribute to lung injury in these patients by quantifying the relationship of EVLW to parameters of lung injury, to determine the effects of chronic alcohol abuse on EVLW, and to determine whether EVLW may be a useful tool in the diagnosis of acute respiratory distress syndrome (ARDS).

**Methods:**

The present prospective cohort study was conducted in consecutive patients with severe sepsis from a medical intensive care unit in an urban university teaching hospital. In each patient, transpulmonary thermodilution was used to measure cardiovascular hemodynamics and EVLW for 7 days via an arterial catheter placed within 72 hours of meeting criteria for severe sepsis.

**Results:**

A total of 29 patients were studied. Twenty-five of the 29 patients (86%) were mechanically ventilated, 15 of the 29 patients (52%) developed ARDS, and overall 28-day mortality was 41%. Eight out of 14 patients (57%) with non-ARDS severe sepsis had high EVLW with significantly greater hypoxemia than did those patient with low EVLW (mean arterial oxygen tension/fractional inspired oxygen ratio 230.7 ± 36.1 mmHg versus 341.2 ± 92.8 mmHg; *P *< 0.001). Four out of 15 patients with severe sepsis with ARDS maintained a low EVLW and had better 28-day survival than did ARDS patients with high EVLW (100% versus 36%; *P *= 0.03). ARDS patients with a history of chronic alcohol abuse had greater EVLW than did nonalcoholic patients (19.9 ml/kg versus 8.7 ml/kg; *P *< 0.0001). The arterial oxygen tension/fractional inspired oxygen ratio, lung injury score, and chest radiograph scores correlated with EVLW (r^2 ^= 0.27, r^2 ^= 0.18, and r^2 ^= 0.28, respectively; all *P *< 0.0001).

**Conclusions:**

More than half of the patients with severe sepsis but without ARDS had increased EVLW, possibly representing subclinical lung injury. Chronic alcohol abuse was associated with increased EVLW, whereas lower EVLW was associated with survival. EVLW correlated moderately with the severity of lung injury but did not account for all respiratory derangements. EVLW may improve both risk stratification and management of patients with severe sepsis.

## Introduction

Severe sepsis is a common syndrome among hospitalized patients, occurring at a rate of 250,000–750,000 cases/year in the USA [1,2]. It is defined as pathologic infection accompanied by a spectrum of physiologic abnormalities, originally described as systemic inflammatory response syndrome criteria in combination with acute organ dysfunction [3,4]. Sepsis is associated with high death rates, killing 30–50% of those severely afflicted [1,5], and is the leading cause of death among patients in noncoronary intensive care units (ICUs) in the USA [6]. According to the annual report from the National Center for Health Statistics [7], sepsis has risen to being the 10th leading cause of death overall in the USA.

Respiratory failure is among the most frequent complications of severe sepsis, occurring in nearly 85% of cases [5]. The mechanisms of acute lung failure in sepsis are complex and incompletely understood [8]. The hallmark of sepsis is increased capillary permeability, which manifests in the lungs as altered alveolar–capillary barrier function and is characterized by accumulation of extravascular lung water (EVLW). However, there is a paucity of data regarding EVLW in patients with severe sepsis.

The most severe form of lung failure, acute respiratory distress syndrome (ARDS), occurs in 40% of patients with sepsis [9]. As with sepsis, ARDS is a heterogeneous clinical syndrome. Recognition of ARDS relies upon a clinical definition, which was standardized in 1994 by the American–European Consensus Conference (AECC) [9]. These criteria comprise a constellation of clinical and radiographic findings that are associated with varying degrees of reliability [10]. No previous diagnostic criteria for ARDS have included measures of EVLW.

A variety of pre-existing comorbid conditions may alter the incidence and severity of ARDS. Chronic alcohol abuse is independently associated with a doubling in risk for developing ARDS, and once ARDS has developed it is associated with a nearly twofold risk for dying [11]. Similarly, chronic alcohol abuse is associated with more severe organ dysfunction in patients with septic shock [12]. Animal models of chronic alcohol abuse confirm the presence of steady-state abnormalities in alveolar–capillary permeability [13]. Initial findings in humans with chronic alcohol abuse suggest that alveolar–capillary barrier function is persistently altered [14].

We hypothesized that acute respiratory failure accompanying severe sepsis relates to subclinical abnormalities in capillary permeability. If this is true, then these abnormalities would be clinically apparent in the accumulation of EVLW across a broad population of patients with severe sepsis. We conducted the largest prospective evaluation to date of EVLW among critically ill patients with severe sepsis. We also evaluated the heterogeneity of EVLW in those patients who developed ARDS and the impact that chronic alcohol abuse had on the accumulation of EVLW and respective outcomes.

## Methods

This study was reviewed and approved by the Institutional Review Board of Emory University School of Medicine. All patients admitted to the Medical ICU at Grady Memorial Hospital between July 2001 and March 2002 were screened for eligibility. Included patients met standard published criteria for severe sepsis [15]. The exclusion criteria were pregnancy, contraindication to femoral artery catheterization, age less than 18 years, and inability to obtain informed consent from the patient or surrogate. All eligible patients were enrolled within 72 hours of meeting criteria for severe sepsis. Patient management decisions, including the type and amount of volume resuscitation, were at the discretion of the primary intensive care physician.

At the time of enrollment, patient-specific data were obtained, including demographic data, past medical and social history, source of sepsis, and Acute Physiology and Chronic Health Evaluation (APACHE) II score [16]. A 5-F arterial catheter (Pulsiocath PV2015; Pulsion Medical Systems, Munich, Germany) was placed in the descending aorta via the femoral artery using the Seldinger technique. The arterial catheter and a standard central venous catheter were connected to pressure transducers and to an integrated bedside monitor (PiCCO; Pulsion Medical Systems). Continuous cardiac output (CO) calibration and EVLW measurements were obtained by triplicate central venous injections of 15–20 ml iced 0.9% saline solution. CO calibrations and determination of EVLW were performed immediately after catheter insertion and at least every 24 hours for 7 days. The catheter system was discontinued before 7 days had elapsed in the event of patient death or transfer from the ICU.

The PiCCO catheter system uses a single thermal indicator technique to determine EVLW, CO, and volumetric parameters. The bolus thermodilution CO is used to determine the patient's aortic impedance, which is used to calibrate the continuous CO [17,18]. CO is calculated using the Stewart–Hamilton method from thermodilution curves measured in the descending aorta, with accuracy comparable with that of pulmonary artery thermodilution [17-21]. The volume of distribution of the thermal indicator represents the intrathoracic thermal volume (ITTV), where ITTV (ml) = CO × mean transit time of the thermal indicator [22,23]. The pulmonary thermal volume (PTV) is given by PTV (ml) = CO × τ, where τ is exponential decay time of the thermodilution curve [24]. Global end-diastolic volume (GEDV), the combined end-diastolic volumes of all cardiac chambers, is given by ITTV – PTV (ml). This permits calculation of intrathoracic blood volume (ITBV) from the linear relationship with GEDV [22,25]: ITBV = 1.25 × GEDV – 28.4 (ml). EVLW is the difference between the thermal indicator distribution in the chest (ITTV) and the blood volume of the chest (ITBV) [22,25-29]: EVLW = ITTV – ITBV (ml).

### Outcome variables

Parameters were indexed to total body surface area or to body weight in order to facilitate comparisons (e.g. EVLW refers to EVLWI). Patients were considered to have elevated EVLW if any measurement was greater than 10 ml/kg, based on previous studies examining the range of EVLW measurements in control patients with no clinical evidence of lung abnormalities [30,31]. Patients were followed for 28 days from enrollment to determine the occurrence of ARDS and death. ARDS was deemed to be present when the AECC criteria [9] were met within 7 days of developing severe sepsis. These criteria are as follows: acute onset of hypoxemia (arterial oxygen tension [PaO_2_]/fractional inspired oxygen [FiO_2_] ratio <200 mmHg) with bilateral infiltrates on chest radiograph and pulmonary artery occlusion pressure ≤ 18 mmHg or no evidence of left atrial hypertension. The severity of ARDS was quantitated using the Lung Injury Score (LIS) [32]. In addition, chest radiograph score (number of quadrants with >50% involvement with an alveolar filling process), PaO_2_/FiO_2 _ratio, and ventilator settings were recorded daily. The lung permeability index was calculated as the ratio of EVLW to ITBV, which was previously shown to reflect permeability of the alveolar–capillary barrier [23,33]. Patients were considered to have a history of chronic alcohol abuse if they had a history of alcohol abuse in their medical records or had a score of at least 3 on the Short Michigan Alcohol Assessment Test [34].

### Statistical analysis

Data are presented as mean ± standard deviation, or as median (interquartile range [IQR]), depending on the distribution normality of the variable. Continuous variable measurements were compared using two-sample t-tests or Mann–Whitney U-tests for normally or non-normally distributed data, respectively. Multiple longitudinal comparisons were made by repeated measures analysis of variance (ANOVA) with time as a covariate. The χ^2 ^statistic was used to compare frequency proportions. Modeling by least squares linear regression for continuous outcome variables and maximum likelihood logistic regression for dichotomous outcome variables was used to assess individual effects while adjusting for individually significant covariables. Statistical analysis was performed using NCSS 2001 software (NCSS, Inc., Kaysville, UT, USA) and all statistical tests were two-sided. *P *= 0.05 was considered statistically significant and *P *> 0.20 is reported as not significant.

## Results

### Severe sepsis study population

Twenty-nine patients with severe sepsis were enrolled at a median of 1 day after development of organ dysfunction requiring ICU admission. Demographic and physiologic characteristics are presented in Table 1. For 17 patients there were complete data for all 7 days; the study was terminated early because of patient death (*n *= 5) or transfer out of the ICU (*n *= 7) in the remaining 12 patients. The sources of sepsis were pneumonia (*n *= 16), intra-abdominal infection (*n *= 6), primary bloodstream infection (*n *= 4), and urosepsis (*n *= 3). The incidence of ARDS, according to the AECC definition, was 52% (15/29). Chronic alcohol abuse was present in 13 out of 29 patients (45%). The overall 28-day mortality was 41% (12/29).

**Figure 1:**
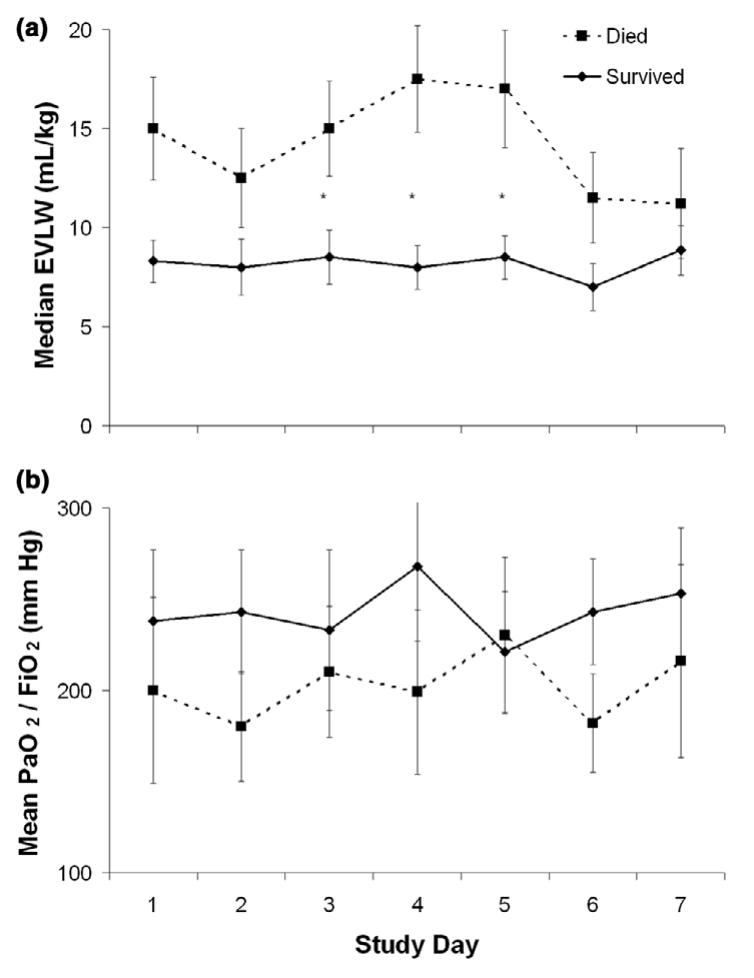
Longitudinal measures of **(a) **extravascular lung water (EVLW) and **(b) **oxygenation (arterial oxygen tension [PaO_2_]/inspired fractional oxygen [FiO_2_] ratio) in patients with severe sepsis, stratified by survival. Vertical bars indicate standard errors. *Significant between-group differences at the marked time points; *P *< 0.001 for EVLW differences over time, by analysis of variance.

At the time of enrollment, the median EVLW for all patients was 8.5 ml/kg (IQR 5.1–15.8 ml/kg). The mean PaO_2_/FiO_2 _ratio was 222.3 ± 149.8 mmHg and LIS was 1.80 ± 1.34; the median chest radiograph score was 2.0 (IQR 1.0–3.0). The mean baseline GEDV index (normal: 680–800 ml/m^2^) was 681 ml/m^2 ^and the mean systemic vascular resistance index (normal: 1800–2500 dyn·s/cm^5 ^per m^2^) was 1528 ± 562 dyn·s/cm^5 ^per m^2^. Fluid balance (net intake/output) was consistently positive, with a cumulative mean during the study period of 8932 ± 9527 ml. The cumulative median EVLW for all patients over time was 9.0 ml/kg (IQR 6.5–15.2 ml/kg) and the mean change in EVLW from the beginning of the study period to the end was -1.1 ± 4.4 ml/kg. EVLW was greater in nonsurvivors than in survivors from severe sepsis (14 ml/kg [IQR 7.4–20 ml/kg] versus 8.0 ml/kg [IQR 5.9–11.2 ml/kg]; *P *< 0.001), and death was associated with greater EVLW over time (Fig. 1a; ANOVA *P *< 0.001). There were no significant longitudinal differences in oxygenation between survivors and nonsurvivors (Fig. 1b).

### Correlates with extravascular lung water

**Figure 2:**
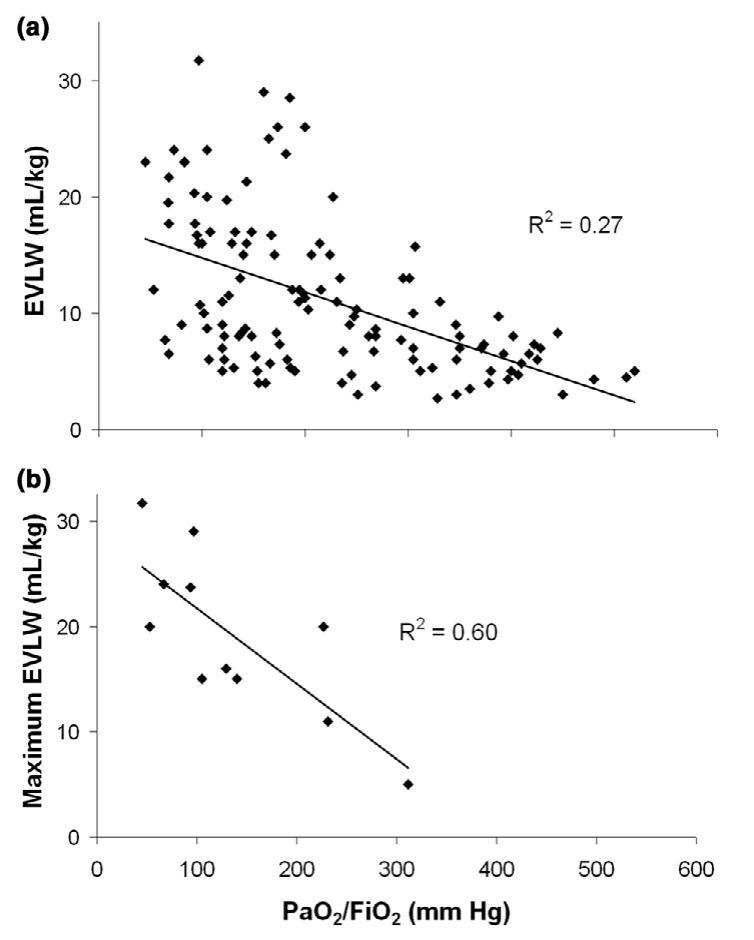
Scatter plot showing the relationship between **(a) **oxygenation (arterial oxygen tension [PaO_2_]/inspired fractional oxygen [FiO_2_] ratio) and extravascular lung water (EVLW) in all patients (R^2 ^by linear regression = 0.27; *P *< 0.001), and **(b) **between minimum PaO_2_/FiO_2 _ratio and maximum EVLW in nonsurvivors (R^2 ^= 0.60; *P *= 0.005).

We examined the relationship between measures of lung injury and EVLW. Using the PaO_2_/FiO_2 _ratio as a measure of oxygenation, we found a statistically significant but moderate correlation with EVLW (r^2 ^= 0.27; *P *< 0.0001; Fig. 2a). Similar relationships were observed between EVLW and the chest radiograph score (r^2 ^= 0.28) and the LIS (r^2 ^= 0.18; both *P *< 0.0001). There was a significant correlation between the highest EVLW and lowest PaO_2_/FiO_2 _ratio (r^2 ^= 0.32; *P *= 0.003), which was greater in nonsurvivors (r^2 ^= 0.60; *P *= 0.005; Fig. 2b) than in survivors (r^2 ^= 0.13; *P *= 0.20). There was a poor correlation between EVLW and GEDV index (r^2 ^= 0.11; *P *< 0.001) and no correlation between EVLW and either daily or cumulative fluid balance.

### Severe sepsis without acute respiratory distress syndrome

The baseline characteristics and physiology of the patients with severe sepsis without ARDS are presented in Table 1; there were no differences in fluid balance or hydrostatic pressure (GEDV index) between this subgroup and all severe sepsis patients combined. The median EVLW for the 14 non-ARDS severe sepsis patients was 7.7 ml/kg (IQR 5.0–10.2 ml/kg), but it was above normal in 57% of patients (8/14; Table 2). The median EVLW for non-ARDS patients with increased EVLW was 12.0 ml/kg (IQR 11.0–14.0 ml/kg), as compared with a median of 6.3 ml/kg (IQR 4.3–8.0 ml/kg) for patients with low EVLW (*P *< 0.001). Non-ARDS patients with a high EVLW were significantly more hypoxic than those with a low EVLW (mean PaO_2_/FiO_2 _ratio 230.7 ± 36.1 mmHg versus 341.2 ± 92.8 mmHg; *P *< 0.001). Calculated LIS values (mean 0.8 ± 0.7 versus 0.6 ± 0.8) and chest radiograph scores (median 2 [IQR 0–2] versus 1 [IQR 0–1]) were not significantly different between the two groups. A statistically insignificant increase in mortality was observed in non-ARDS patients with high EVLW (50% versus 17%; *P *= 0.20).

### Severe sepsis with acute respiratory distress syndrome

Baseline characteristics and physiology for severe sepsis patients who developed AECC-defined ARDS (*n *= 15) were similar to those for the non-ARDS patients, with the exception of greater EVLW (Table 1) and increased measures of lung permeability (lung permeability index [EVLW/ITBV ratio] 1.18 ± 0.45 versus 0.60 ± 0.31; *P *< 0.001). Fluid balance and hydrostatic pressures were not different at baseline or longitudinally from those in non-ARDS patients, and did not correlate with the development of ARDS. GEDV index correlated weakly with EVLW (r^2 ^= 0.17; *P *< 0.001) whereas fluid balance did not correlate. Differences according to EVLW for the ARDS patients are presented in Table 2. The median EVLW for ARDS patients was 12.0 ml/kg (IQR 7.8–17.7 ml/kg) and the diagnosis of ARDS was associated with increased EVLW over time compared with non-ARDS patients (repeated measures ANOVA, *P *< 0.001).

Of the ARDS patients, only 73% (11/15) had any evidence of increased EVLW during the study period. The median EVLW for ARDS patients with low EVLW patients was 7.0 ml/kg (IQR 6.0–8.3 ml/kg), as compared with 16.9 ml/kg (IQR 14.8–22.3 ml/kg) for the high EVLW ARDS patients (*P *< 0.001). Cumulative mean oxygenation during the study period was worse among high EVLW ARDS patients (PaO_2_/FiO_2 _ratio 135.4 ± 60.4 versus 197.0 ± 106.7 mmHg; *P *= 0.001). Cumulative mean chest radiograph scores (4 [IQR 4–4] versus 3 [IQR 2–4]; *P *= 0.002) and LIS (2.8 ± 1.1 versus 2.1 ± 0.7; *P *= 0.002) were similarly worse in high EVLW ARDS patients.

There was significantly reduced mortality among the 27% of ARDS patients with consistently low EVLW as compared with the ARDS patients with high EVLW (0/4 versus 7/11; *P *= 0.03). The high EVLW group had a significantly greater APACHE II score than did the low EVLW group (25.9 ± 6.3 versus 18.5 ± 3.3; *P *= 0.05), although differences in APACHE II score accounted for under 10% of the differences in EVLW by univariate regression analysis. If EVLW were substituted for bilateral radiographic infiltrates in the AECC diagnostic criteria, then three additional patients would have been diagnosed with ARDS, increasing the incidence by 20%.

### Chronic alcohol abuse

Chronic alcohol abuse was present in 45% (13/29) of the severe sepsis patients, including 33% (5/15) of ARDS patients (Table 3). Patients with alcohol abuse had no evidence of cirrhosis or ascites. Hydrostatic pressures and serum albumin levels were not different from those in nonalcoholic patients. The lung permeability index was increased in ARDS patients with chronic alcohol abuse as compared with nonalcoholic ARDS patients (1.73 ± 0.33 versus 1.20 ± 0.47; *P *= 0.04). Net fluid intake was greater in the 24 hours before enrollment in alcoholic patients with ARDS (Table 3), although cumulative fluid balance during the study period was not different (10683 ± 10247 ml versus 7415 ± 8929 ml; not significant). Adjustment for baseline differences in fluid balance by linear regression revealed that alcohol abuse independently predicts greater EVLW by an average of 9.3 ml/kg in ARDS patients (*P *< 0.001).

All five ARDS patients with a history of chronic alcohol abuse had increased EVLW. Among ARDS patients, the chronic alcoholic patients' median EVLW over the course of the study was significantly elevated as compared with that in nonalcoholic patients (19.9 [IQR 16.0–28.5] ml/kg versus 8.7 [IQR 7.7–11.0] ml/kg; *P *< 0.0001); a similar relationship existed for non-ARDS patients (median alcoholic EVLW 8.7 [IQR 5.0–10.3] ml/kg versus 7.0 [IQR 5.0–8.0] ml/kg; *P *= 0.04). The relative risk for high EVLW was 2.4 times greater in ARDS patients with chronic alcohol abuse (*P *= 0.03). Using a repeated measures ANOVA, chronic alcohol abuse was associated with higher EVLW over the 7-day study duration among all patients (*P *= 0.04) and the subset of ARDS patients (*P *< 0.001). Mortality was 54% (7/13) for chronic alcoholic patients versus 31% (5/16) for nonalcoholic patients (not significant).

## Discussion

Among severe sepsis patients without clinical ARDS, more than half manifest abnormal quantities of EVLW. Despite not meeting the consensus conference definition for ARDS, the amount of EVLW correlated with measures of lung injury (PaO_2_/FiO_2 _ratio, LIS, and chest radiograph score). Half of these patients were adequately hypoxemic to diagnose ARDS by the AECC criteria, but they did not exhibit the necessary bilateral radiographic infiltrates. Furthermore, 27% of the patients fulfilling the clinical consensus conference criteria for ARDS never had elevated EVLW, and these patients had improved survival as compared with ARDS patients with increased EVLW. These data support the hypothesis that EVLW varies substantially among patients with severe sepsis, and thus it may contribute to the high frequency of respiratory dysfunction. In addition, we found that severe sepsis patients with a history of chronic alcohol abuse had significantly greater EVLW than did nonalcoholic patients. This relationship was strengthened by the presence of ARDS, thus demonstrating the importance of comorbid disease for the risk and severity of ARDS.

Our findings have both diagnostic and prognostic implications for patients with severe sepsis. EVLW parallels the common clinical pathway and represents the physiologic derangements of ARDS, but it is not included in the AECC definition. Given that accumulation of lung water is one of the hallmarks of ARDS, the fact that 57% of severe sepsis patients without clinical ARDS have increased EVLW suggests that these patients have an unrecognized form of lung injury. Thus, despite the presence of hypoxemia, the AECC definition for ARDS may be insensitive to more subtle forms of ARDS because of variability in interpretation of chest radiograph [35] and the greater sensitivity of EVLW measures for detecting pulmonary edema [36,37]. Similar concerns have been voiced about the specificity of the definition [10], highlighting the need for an accurate early diagnostic marker when the diagnosis may be uncertain and therapeutic interventions may be most critical.

EVLW additionally serves as a prognostic marker for patients with ARDS. Previous studies have estimated EVLW in states of respiratory failure and/or ARDS with conflicting outcome results [38-42]. Modern studies including strictly defined ARDS patients corroborate an effect on mortality, particularly if changes in EVLW are considered over time [38]. However, historical methods of estimating EVLW have been complex, clinically difficult, and poorly reproducible [36,43-46]. The most common method of estimating EVLW continues to be with chest radiography, despite being imprecise and highly variable [36,37,47]. Given the ready availability and relative simplicity of EVLW measures compared with past methods, additional clinical trials are warranted to compare EVLW as a prognostic marker with other modern standards, such as pulmonary dead space [48].

The implications of EVLW measurements for severe sepsis patients with a history of chronic alcohol abuse may be even greater. The rate of development of ARDS among critically ill chronic alcoholic individuals is twice that in nonalcoholic individuals [11]; the risk is even higher among chronic alcoholic patients with severe sepsis (relative risk = 2.43, 95% confidence interval = 1.55–3.86). [12] The underlying mechanisms for increased ARDS susceptibility in chronic alcoholic individuals involve permeability defects, in which animal models of alcoholism have shown altered alveolar–capillary membrane permeability [13]. The mechanism for this alteration arises from perturbations in glutathione homeostasis, with otherwise healthy chronic alcoholic individuals having reduced levels of glutathione in their alveolar epithelial lining fluid [49] and apparent increased permeability to proteins [14]. The present report is the first to show an exaggerated increase in EVLW among chronic alcoholic ARDS patients, correlated with measures reported to indicate lung capillary permeability (lung permeability index), supporting the hypothesis that an ineffective permeability barrier may predispose susceptible alcoholic patients to heightened development of ARDS.

This study has several limitations. The size of the study prevents absolute conclusions from being drawn regarding EVLW in patients with severe sepsis, although these results stand as the largest prospective evaluation of EVLW in patients with severe sepsis. The transpulmonary thermodilution technique employed for measuring EVLW has been well validated in critically ill patients [22,25,38,50] despite prior concerns that severe ventilation–perfusion mismatch may preclude access to the complete pulmonary vascular bed [51]. All chest radiographs were interpreted by a single experienced critical care physician to reduce variability in interpretation of chest radiographs [35]. The apparent insensitivity of the consensus ARDS definition may be improved with consideration of less severe forms of lung injury, although this is operationally differentiated by the severity of hypoxemia rather than the discrepant factor in our study, namely evidence of pulmonary edema on chest radiograph. The finding that ARDS patients with higher EVLW have increased mortality, as well as the finding of no difference in mortality among severe sepsis patients stratified by EVLW, may be due to statistical power or inherent heterogeneity in the sepsis and ARDS patient populations (beyond such identified disparities as baseline fluid balance).

## Conclusion

Lung water accumulates abnormally in a substantial fraction of severe sepsis patients without recognized respiratory complications. These subtle abnormalities of pulmonary function may represent subclinical lung injury, which are undetectable by standard techniques and current clinical definitions. Furthermore, EVLW has prognostic implications for patients with severe sepsis and ARDS, and correlates with the severity of lung injury. More importantly, EVLW is highly prognostic for critically ill patients with chronic alcohol abuse, presumably representing intrinsic altered alveolar–capillary integrity. Further investigation is required to confirm these findings and to determine the utility of EVLW as a diagnostic or prognostic marker in patients with severe sepsis.

## Key messages

• 	The majority of severe sepsis patients have increased amounts of EVLW, including those who do not meet clinical criteria defining ARDS.

• 	Increased EVLW is associated with worse survival in patients with severe sepsis, whereas the minority of ARDS patients with normal amounts of EVLW have greater chances of survival.

• 	Chronic alcohol abuse is associated with increased quantities of EVLW, presumably reflecting inherent alveolar–capillary barrier dysfunction.

• 	Measurements of EVLW may serve to risk stratify severe sepsis patients and to improve patient management.

## Abbreviations

AECC = American–European Consensus Conference; ANOVA = analysis of variance; APACHE = Acute Physiology and Chronic Health Evaluation; ARDS = acute respiratory distress syndrome; CO = cardiac output; EVLW = extravascular lung water; FiO_2 _= fractional inspired oxygen; GEDV = global end-diastolic volume; ICU = intensive care unit; IQR = interquartile range; ITBV= intrathoracic blood volume; ITTV = intrathoracic thermal volume; LIS = Lung Injury Score; PaO_2 _= arterial oxygen tension; PTV = pulmonary thermal volume.

## Competing interests

The author(s) declare that they have no competing interests.

## Authors' contributions

GM was involved in the study concept and design; collection, analysis and interpretation of the data; provision of study materials and patients; statistical expertise; obtaining funding; and drafting, revision, and approval of the manuscript. SE was involved in the collection, analysis, and interpretation of the data; provision of study materials and patients; and drafting, revision, and approval of the manuscript. MM (Mealer) was involved in the collection, analysis, and interpretation of the data; provision of study materials and patients; and approval of the manuscript. MM (Moss) was involved in study concept and design; collection, analysis, and interpretation of the data; provision of study materials and patients; statistical expertise; and drafting, revision, and approval of the manuscript.

## Figures and Tables

**Table 1:** Baseline demographic characteristics and outcomes for all patients with severe sepsis

	ARDS	Non-ARDS	Total
Patients (*n*)	15	14	29
Baseline characteristics			

Age (years)	44 ± 12	57 ± 14	51 ± 15*
Sex (male)	5/15	5/14	10/29
Race (African American/Caucasian/other)	12/3/0	12/0/2	24/3/2
Chronic heart disease	1/15	2/14	3/29
Chronic obstructive lung disease	1/15	1/15	2/29
Chronic renal disease (serum creatinine ≥ 2.0 mg/dl)	1/15	3/14	4/29
HIV infection	7/15	4/14	11/29
Chronic alcohol abuse	5/15	8/14	13/29
Bacteremia	7/15	5/14	12/29
APACHE II score (day 1)	23.9 ± 6.5	27.7 ± 8.3	25.7 ± 7.5
Mechanically ventilated	14/15	11/14	25/29
Physiology at enrollment			

White blood cell count (cells × 10^3^/ml)	20.4 (10.8–30.0)	13.6 (3.8–23.4)	17.2 (8.9–25.5)
Prior 24-hour fluid balance (ml)	2506 ± 2583	4993 ± 3625	3523 ± 3223
Shock (vasopressor requirement)	12/15	10/14	22/29
Cardiac index (l/min per m^2^)	3.8 ± 1.0	4.3 ± 1.4	4.0 ± 1.2
GEDV index (ml/m^2^)	648 ± 184	719 ± 119	681 ± 159
SVRI (dyn·s/cm^5 ^per m^2^)	1408 ± 441	1668 ± 669	1528 ± 562
EVLW index (ml/kg)	15.0 (9.0–16.7)	7.0 (5.0–8.2)	8.5 (5.1–15.8)*
Outcome			

ICU length of stay (days)	14.0 (8.5–21.0)	10.0 (4.8–16.3)	13.0 (7.0–17.0)
Hospital length of stay (days)	26.0 (12.3–42.3)	19.0 (16.0–31.0)	19.0 (13.0–36.0)
28-day mortality	7/15	5/14	12/29

Values are expressed as mean ± standard deviation, frequency (%), or median (interquartile range), unless otherwise noted.

* *P *for difference between groups < 0.05. APACHE, Acute Physiology and Chronic Health Evaluation; ARDS, acute respiratory distress syndrome; EVLW, extravascular lung water; GEDV, global end-diastolic volume; ICU, intensive care unit; SVRI, systemic vascular resistance index.

**Table 2:** Demographics, baseline patient characteristics and outcomes of ARDS and non-ARDS patients stratified by lung water content

	Non-ARDS			ARDS		
	
	Low EVLW (*n *= 6)	High EVLW (*n *= 8)	*P*	Low EVLW (*n *= 4)	High EVLW (*n *= 11)	*P*
EVLW (ml/kg)	5.0 (4.8–8.1)	7.7 (5.0–8.7)	NS	9.0 (8.0–9.8)	16.0 (13.0–20.3)	0.07
Source (*n *[% pulmonary])	2/6 (33%)	3/8 (38%)	NS	3/4 (75%)	8/11 (73%)	NS
APACHE II score	27.8 ± 2.3	27.6 ± 11.6	NS	18.5 ± 3.3	25.9 ± 6.3	0.05
PaO_2_/FiO_2 _index (mmHg)	424.0 ± 51.7	244.3 ± 133.9	0.02	91.5 ± 24.6	144.9 ± 89.9	NS
LIS	0.8 ± 0.2	0.9 ± 0.9	NS	2.6 ± 1.7	2.7 ± 1.1	NS
CXR score	0.5 (0–1)	1 (0–2)	NS	3 (2–4)	4 (3–4)	NS
GEDV index (ml/m^2^)	750.5 ± 133.6	691.3 ± 108.1	NS	581.5 ± 131.4	671.8 ± 200.0	NS
Fluid balance (prior 24 hours; ml)	5256 ± 4918	4664 ± 1591	NS	2373 ± 776	2545 ± 2959	NS
Chronic alcohol abuse (*n *[%])	4/6 (67%)	4/8 (50%)	NS	0/4 (0%)	5/11 (45%)	0.09
28-Day mortality (*n *[%])	1/6 (17%)	4/8 (50%)	0.20	0/4 (0%)	7/11 (64%)	0.03

Extravascular lung water (EVLW), Acute Physiology and Chronic Health Evaluation (APACHE) II, arterial oxygen tension (PaO_2_)/fractional inspired oxygen (FiO_2_) ratio, Lung Injury Score (LIS), and chest radiographic (CXR) score are initial values calculated from study day 1. Values are expressed as means ± standard deviation or as median (interquartile range). ARDS, acute respiratory distress syndrome.

**Table 3:** Demographics, baseline patient characteristics and outcomes of ARDS and non-ARDS patients stratified by history of chronic alcohol abuse

	Non-ARDS			ARDS		
	
	Chronic alcohol abuse (*n *= 8)	Nonalcoholic (*n *= 6)	*P*	Chronic alcohol abuse (*n *= 8)	Nonalcoholic (*n *= 6)	*P*
EVLW (ml/kg)	7.0 (5.0–8.0)	6.5 (4.8–8.8)	NS	20.3 (16.0–26.7)	9.5 (7.1–15.4)	0.009
Source (*n *[% pulmonary])	2/8 (25%)	1/6 (17%)	NS	5/5 (100%)	7/10 (70%)	0.17
APACHE II score	28.7 ± 9.3	26.5 ± 7.7	NS	27.0 ± 6.7	22.4 ± 6.1	NS
PaO_2_/FiO_2 _index (mmHg)	369.7 ± 140.8	215.7 ± 116.7	NS	96.7 ± 21.7	148.0 ± 95.1	NS
LIS	0.9 ± 0.6	0.8 ± 0.7	NS	3.2 ± 0.5	2.4 ± 1.4	NS
CXR score	0.5 (0.0–1.3)	1.0 (0.5–1.5)	NS	4.0 (2.5–4.0)	3.5 (2.5–4.0)	NS
GEDV index (ml/m^2^)	750.3 ± 142.7	681.7 ± 81.6	NS	714.7 ± 149.7	614.2 ± 197.9	NS
Fluid balance (prior 24 hours; ml)	4703 ± 3310	6004 ± 6064	NS	4758 ± 3240	1504 ± 1550	0.03
28-Day mortality (*n *[%])	4/8 (50%)	1/6 (17%)	0.19	3/5 (60%)	4/10 (40%)	NS

Extravascular lung water (EVLW), Acute Physiology and Chronic Health Evaluation (APACHE) II, arterial oxygen tension (PaO_2_)/fractional inspired oxygen (FiO_2_) ratio, Lung Injury Score (LIS), and chest radiographic (CXR) score are initial values calculated from study day 1. Values are expressed as means ± standard deviation or as median (interquartile range). ARDS, acute respiratory distress syndrome.
